# Genome wide prediction of protein function via a generic knowledge discovery approach based on evidence integration

**DOI:** 10.1186/1471-2105-7-268

**Published:** 2006-05-25

**Authors:** Jianghui Xiong, Simon Rayner, Kunyi Luo, Yinghui Li, Shanguang Chen

**Affiliations:** 1 Laboratory of Space Cell and Molecular Biology, China Astronaut Research and Training Center, Beijing, P.R. China; 2 Wuhan Institute of Virology, Chinese Academy of Sciences, Wuhan, Hubei, P.R. China; 3 Laboratory of Space Computer Simulation, China Astronaut Research and Training Center, Beijing, P.R. China

## Abstract

**Background:**

The automation of many common molecular biology techniques has resulted in the accumulation of vast quantities of experimental data. One of the major challenges now facing researchers is how to process this data to yield useful information about a biological system (e.g. knowledge of genes and their products, and the biological roles of proteins, their molecular functions, localizations and interaction networks). We present a technique called Global Mapping of Unknown Proteins (GMUP) which uses the Gene Ontology Index to relate diverse sources of experimental data by creation of an abstraction layer of evidence data. This abstraction layer is used as input to a neural network which, once trained, can be used to predict function from the evidence data of unannotated proteins. The method allows us to include almost any experimental data set related to protein function, which incorporates the Gene Ontology, to our evidence data in order to seek relationships between the different sets.

**Results:**

We have demonstrated the capabilities of this method in two ways. We first collected various experimental datasets associated with yeast (*Saccharomyces cerevisiae*) and applied the technique to a set of previously annotated open reading frames (ORFs). These ORFs were divided into training and test sets and were used to examine the accuracy of the predictions made by our method. Then we applied GMUP to previously un-annotated ORFs and made 1980, 836 and 1969 predictions corresponding to the GO Biological Process, Molecular Function and Cellular Component sub-categories respectively. We found that GMUP was particularly successful at predicting ORFs with functions associated with the ribonucleoprotein complex, protein metabolism and transportation.

**Conclusion:**

This study presents a global and generic gene knowledge discovery approach based on evidence integration of various genome-scale data. It can be used to provide insight as to how certain biological processes are implemented by interaction and coordination of proteins, which may serve as a guide for future analysis. New data can be readily incorporated as it becomes available to provide more reliable predictions or further insights into processes and interactions.

## Background

Advances in DNA sequencing technology in recent years has seen the completion of a large number of genomes, with the completion of many more planned for the future. However, the generation of a DNA sequence map is only the first step in obtaining an understanding of an organism or species. One of the main goals of the post-genomic era is to obtain knowledge of genes and gene products, such as the biological roles of proteins, their molecular functions, localizations and their interaction networks in living organisms.

In the past, protein function would be determined by an experimental investigation of activity and quantification of abundances in specific locations. However, with the sheer quantity of data awaiting processing, this method of classification alone is no longer sufficient and more automated large scale methods of experimental analysis are required. Examples of these techniques include microarray gene expression profiles [1-4], protein interactions revealed by yeast two-hybrid system [5,6] and protein complexes identification by mass spectrometry[7,8]. While these methods have all been successful in the characterization of biological systems, they have in turn generated additional large quantities of data which also require analysis to extract useful information. Many software tools have been developed to aid the scientist in mining these data to identify features and defining characteristics. The existing genome-scale protein function prediction methods currently in use can be (roughly) grouped into three categories:

1. Methods based on sequence or protein characteristics. The most common of these are tools such as FASTA [9] and PSI-BLAST [10]. Several non-homology-based methods have also been introduced, e.g. the Rosetta Stone [11,12], the phylogenetic method [13], the chromosomal proximity method [14] and the combined method [15].

2. Methods based on mining single types of genome-scale data. Examples of this data includes microarray gene expression profiles, yeast two-hybrid systems and protein complexes using established techniques such as clustering analysis [16,17], or alternative methods such as the shortest-path approach [18], the method based on overlapping transcriptional clusters [19] and temporal gene expression patterns [20].

3. Methods based on integration of heterogeneous data formats. Since valuable information also exists in relationships between aspects of data existing in different datasets, several data integration and mining methods have been introduced to utilize these relationships when predicting proteins function. These include formal Bayesian reasoning [21], e.g. Bayesian statistical methods combined with a Boltzmann machine and simulated annealing [22,23] and a Hopfield network approach to integrate gene expression and protein interaction data [24,25].

While many of the analytic methods outlined in classifications 1 and 2 have been successfully applied to identify unknown functions for protein, it is the third classification of analysis techniques which has the most potential for identification of function (and possibly novel relationships) for unknown proteins. This is because these methods collate experimental information from an eclectic range of sources before making any predictions. However, two of the major complications faced by this type of analysis are the (i) variation in terminology that exists between different datasets and (ii) the differences in storage format. These disparities can make discoveries of even simple correlations a formidable challenge and may result in important relationships being overlooked.

The Gene Ontology Consortium (GO) [26] attempts to address these types of problems by providing a method by which different datasets may be related via a common set of definitions. The GO defines a number of vocabularies to allow classification of gene properties with a degree of precision that may be defined by the end user. For example, a gene involved in a cellular process may simply be defined in these terms, or, if more information is known, a more specific definition might be given as "*cellular process->cellular physiological process->cellular metabolism*". Thus, data from two different sources which relate to the cellular process may be linked via this vocabulary and used together to permit a more detailed analysis or definition of the process. Within the GO, gene properties may be defined via three vocabularies which specify biological process (BP), molecular function (MF) and cellular component (CC). Thus, the GO provides a means of correlating data from a wide range of sources to generate a much broader data set for function prediction.

**Figure 1:**
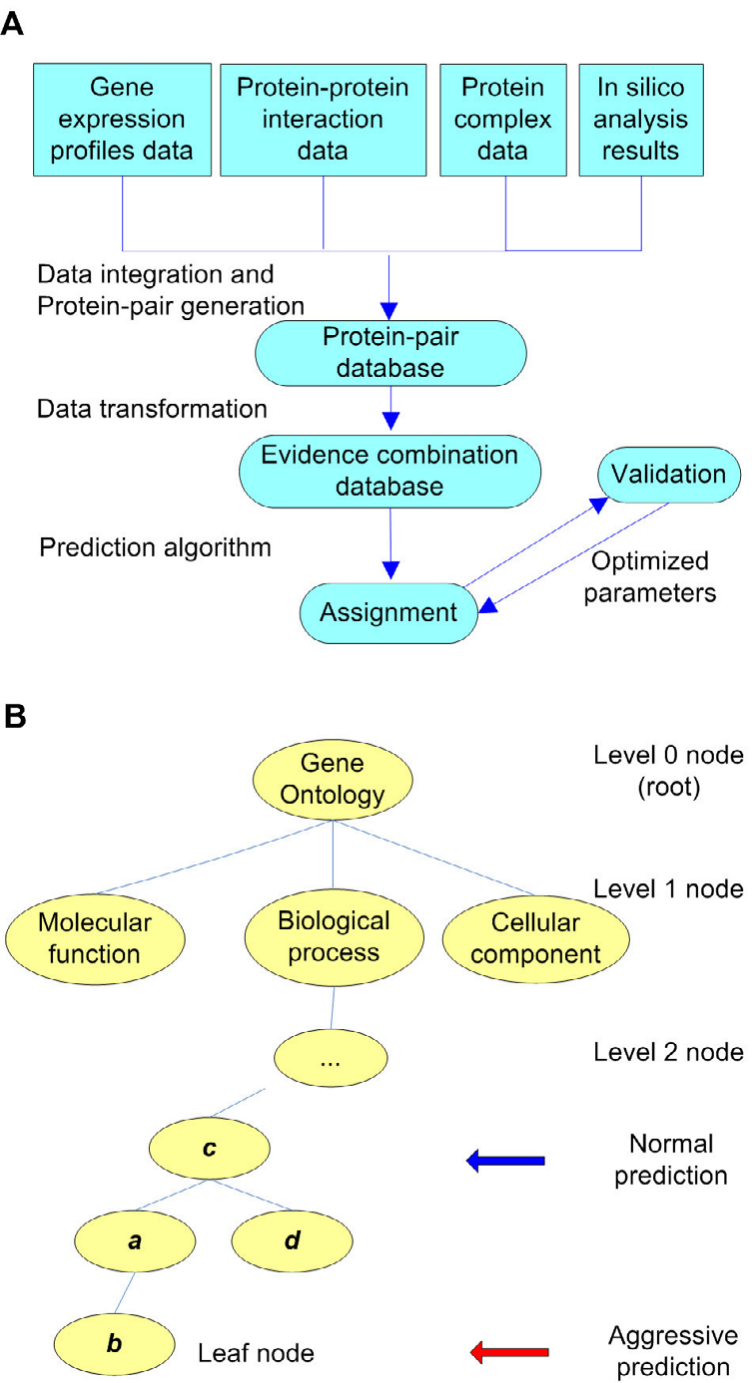
**A schematic illustration of prediction rationale applied in GUMP**. **(A) **Overview of the data integration and the validation process. **(B) **GO structure and the definition of normal prediction and aggressive prediction. Normal Prediction seeks matches to less specific nodes (such as node c) whereas Aggressive Prediction seeks more specific nodes further down the graph (such as node b). Less specific nodes selected by Normal Prediction are generally more reliable since more data is associated with these nodes.

Here we propose a method which addresses many of these issues by firstly relating a broad range of experimental data sets via the GO index and secondly by using a neural network to mine the combined information to imply novel protein function through identified relationships (Fig. 1a). We have demonstrated the capabilities of this method by applying it to several experimental data sets associated with the yeast genome and have identified several novel relationships between unknown proteins and annotated protein complexes.

## Results

### Model evaluation by applying cross-validation on known annotations

Our training and evaluation data was generated from a set of 6167 yeast ORFs with known GO and SGD annotation in the ratio of 9: 1. After network training we evaluated the precision of the network predictions using the evaluation data. Since the evaluation data also consisted of ORFs with known function we were able to evaluate the consistency of the predictions with the original annotation and the trade-off between precision and the sensitivity of predictions. This data could also provide useful feedback for improving the prediction performance of the neural networks (see *METHODS *– *Model Validation and Parameter Optimization*). We also compared the performance of the network when using Aggressive Prediction and Normal Prediction (Fig. 1b, See *METHODS *– *Prediction Model *for a description of the differences between these prediction models). For each different GO vocabulary (biological process, molecular function and cellular component), we trained and validated two neural networks; one for Aggressive Prediction mode and one for Normal Prediction mode (to create a total of six neural networks).

**Figure 2:**
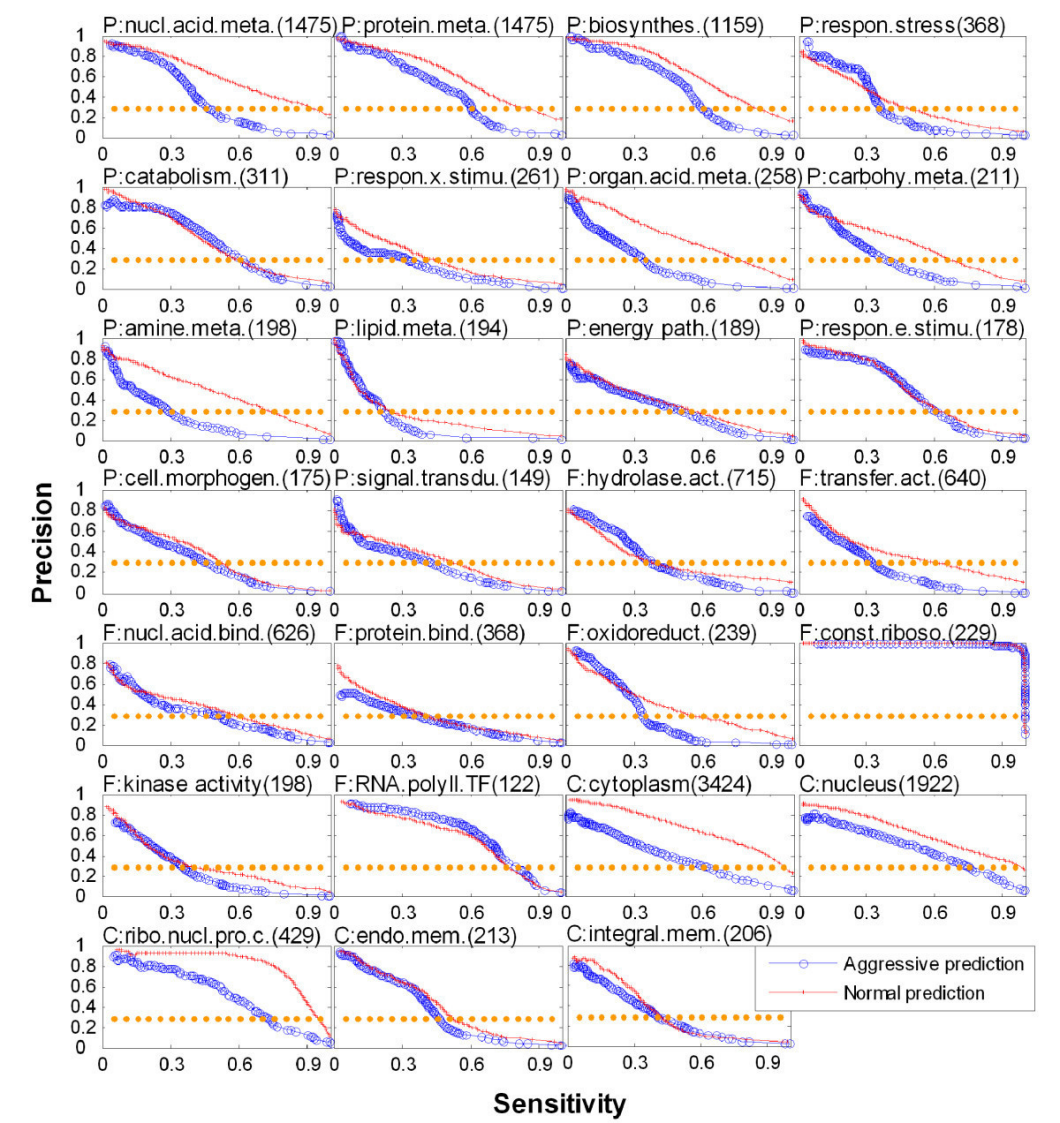
**Validation of predictions using known annotations in diverse subcategories in GO**. The prediction performance is shown here on a total of 27 GO subcategories: 14 for BP, eight for MF, and five for CC subcategories (see **Methods**). In each panel, the title is the name of GO subcategory with a label prefix "P", "F", or "C" to denote biological process, molecular function, or cellular component, respectively. The number in parentheses indicates the total number of ORFs annotated in each GO subcategory. A plot of precision (y-axis) as a function of sensitivity ratio is shown for all unknown ORFs (x-axis), as defined in **Methods**. In such plots, the upper right corner corresponds to a perfect predictor because of the larger area under the precision–sensitivity curve.

Compared with aggressive prediction, normal prediction returned a consistently better prediction performance in almost all subcategories (Fig. 2). This is not unreasonable since this method assigns function according to higher level, less specific nodes on a GO graph which tend to be statistically more reliable.

The best predictions were achieved for two closely related subcategories: the structural constituent of the ribosome (molecular function) and the ribonucleoprotein complex (cellular component). This prediction is consistent with the results both from analyses of gene expression data [2-4] and from other studies involving the integration of additional datasets [21]. This is possibly because the expression results for the ribosome consist of clusters of highly correlated genes which are readily identified by the network.

**Figure 3:**
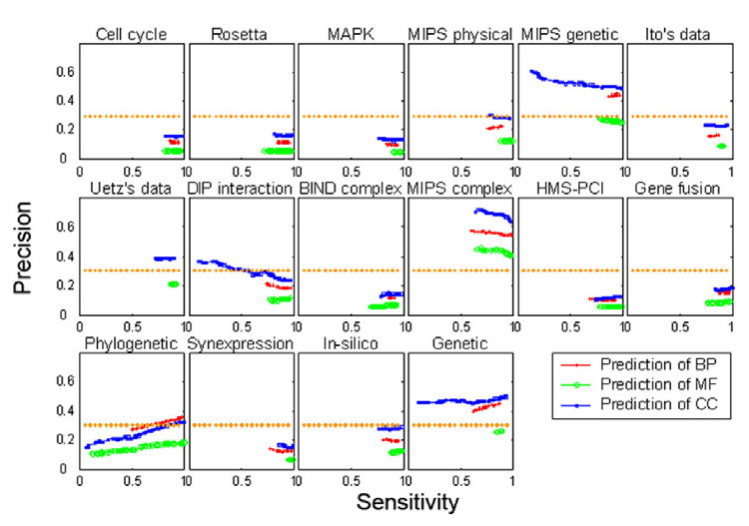
**The relative contribution of different evidence sources to prediction performance**. This figure shows the contribution from individual data sets when input individually to the neural network. The plot axes are the same as **Figure 2**. The most reliable predictions come from MIPS data corresponding to physical interaction, genetic interaction and complex and gene expression profile data (cell-cycle data, the Rosetta Compendium, and MAPK data).

We also examined the ability of the network to predict function using only a single data source. While the complexity of the network topology obviates such a simplistic analysis of the contribution of each data source to the final prediction, it does provide some indication of the bias of the information within each set. It was found that the best results were achieved with three types of MIPS data [27]: physical interaction, genetic interaction and complex (Fig. 3), which are commonly considered by the yeast research community to represent the most reliable data sets. For gene expression profile data, three evidence sources: cell-cycle data, the Rosetta Compendium, and MAPK data, demonstrated that mRNA co-expression data have greater ability to predict cellular component and biological process than to predict molecular function. This is in agreement with many previous genome-wide analyses, which demonstrated that mRNA transcript expression patterns or co-regulation are similar for groups of functionally related genes and subcellular protein localizations [23,28,29].

### Using the model to predict GO annotation of unknown proteins

**Figure 4:**
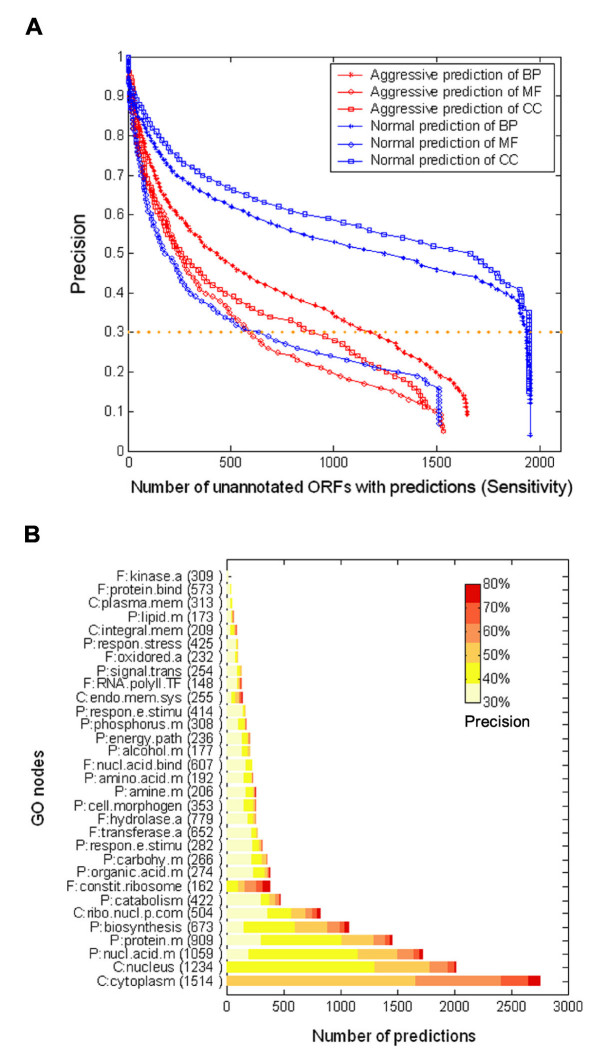
**Gene Ontology predictions for previously un-annotated ORFs in yeast**. **(A) **Trade-off between anticipated conservative precision and sensitivity. The anticipated conservative precision (y-axis) and the expected number of previously un-annotated ORFs with predictions (x-axis) are plotted by modulating parameters such as k (see **Methods**). **(B) **Normal predictions for previously un-annotated ORFs are shown for 31 subcategories for which validations indicate a conservative precision of ≥ 0.3. The subcategories (vertical axis) were sorted by descending number of predictions for conservative precision is larger than 0.3. The colour map reflects the conservative precision of predictions made in each GO subcategory. Red indicates more reliable predictions, yellow represents less reliable predictions. The number in parentheses indicates the total number of predictions made in each subcategory.

Once we had trained and optimized the network, we applied it to a set of previously un-annotated yeast ORFs. Based on the trade-off between precision and sensitivity of the predictions shown in Fig. 2, we selected a precision threshold of 0.3 as an acceptable level for predictions. Using this threshold, we produced predictions for 2304, 2932, and 2169 un-annotated ORFs in the BP [see Additional file 6], MF [see Additional file 7], and CC [see Additional file 8] annotation categories and made 1980, 836, and 1969 predictions respectively (Fig. 4, see also *METHODS *– *Prediction of Uncharacterized Genes*). The Sensitivity-Precision curve for all the predictions is shown in Figure 4a. A complete list of all the predictions is available [see Additional file 5]. A graph of the genes with the most predictions and their associated precision is shown in Figure 4b. The cellular component categories that have the most predictions and best precision (greater than 80%) are the cytoplasm and nucleus. For biological process, nucleic acid metabolism, protein metabolism, and biosynthesis all principally occur in the cytoplasm and are also well represented in the predictions. Once again, this is also consistent with other systematic analyses of gene expression profiles and cellular processes related to metabolism in the cytoplasm [2-4].

The MIPS database provides information such as functional category, protein classification, and localization category, which are similar to the biological process, molecular function, and cellular component, respectively, in the GO index. We therefore compared our predictions with annotations within the MIPS database. We found that our predictions generally agreed with those from MIPS (Table 1, [see Additional file 1] for the complete list and [see Additional file 5] for detailed description). We found that our predictions generally agreed with those of previous studies aiming to predict GO annotations [19,25]. However, our results generally provide more detailed information for protein function. Also, in many cases, we had multiple predictions to nodes that were related by a parent-child relationship. In these situations the more specific predictions of the child node were supported by the related parent assignments which further improved the confidence of our predictions.

In addition, we made 472 higher quality predictions (precision >= 0.8) corresponding to 154 ORFs which have been assigned new or updated GO annotation in the latest SGD release [see Additional file 9]. We compared our 472 predictions with their corresponding GO annotation by calculating the shortest distance on the GO graph from our prediction to the GO node assigned in the release. We found that GMUP was particularly successful at predicting ORFs with functions associated with the ribonucleoprotein complex, protein metabolism and transportation [see Additional file 4]. Based on this, we made a cautious prediction for the function of several of the remaining un-annotated ORFs, which gave 624 predictions for 232 ORFs at an acceptable precision level (>= 0.6, [see Additional file 10]).

### Generating a glimpse of how biological processes are implemented by the interactions of proteins

**Figure 5:**
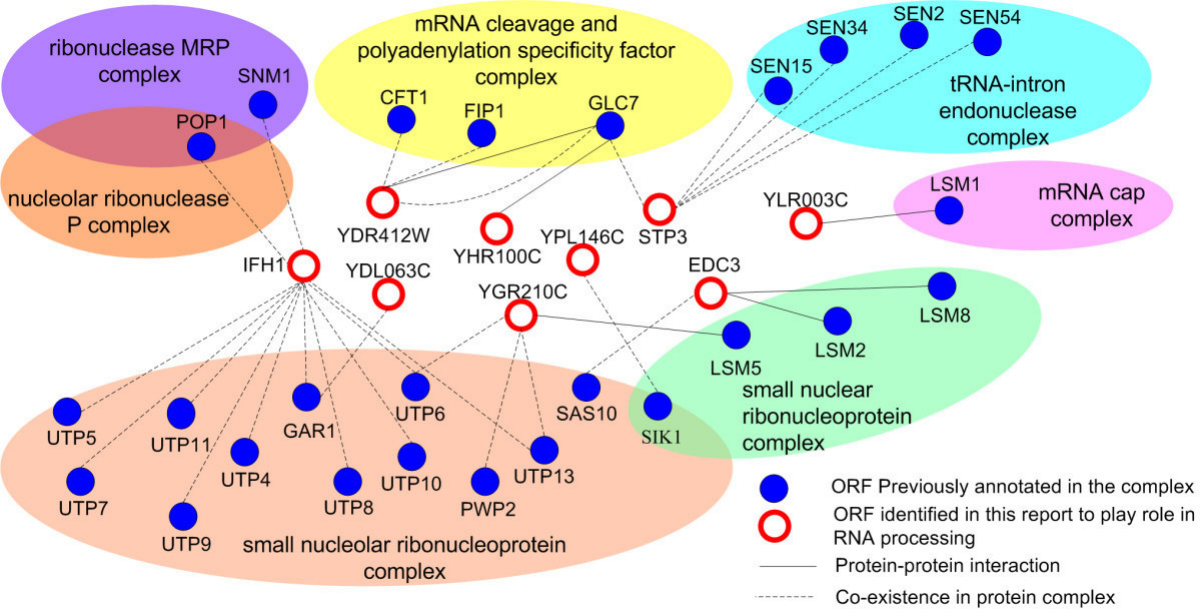
**Schematic representation of proteins contributing to RNA processing and their interaction network**. Proteins previously annotated to certain protein complexes and that have a role in RNA processing are shown in blue, and proteins assigned in this report are white, outlined in red. The protein-pair identified by protein–protein interactions are represented as black lines, and those identified by their co-existence in a protein complex as dashed black lines. New assigned proteins or ORFs were fetched by a query using a conservative precision ≥ 0.7. For figure clarity, the count of protein-pair between two proteins is not shown here (one protein-pair is often supported by multiple evidences in our dataset).

To demonstrate the application of GMUP to the discovery of unknown ORF function, we ran a query to fetch assigned unknown ORFs that are predicted to have a role in RNA processing (biological process annotation), and found a number of proteins with interaction or co-existence relationships with complexes that have been identified to contribute to RNA processing (Fig. 5). We identified *STP3 *as having a strong interaction with the tRNA-intron endonuclease complex (cellular component) and a role in the tRNA splicing process (biological process). This agrees well with its MIPS annotation and SGD description: "*involved in pre-tRNA splicing and in the uptake of branched-chain amino acids*". Another example was *IFH1*, which was annotated to the nucleus [30,31] by the evidence string "*inferred from direct assay *(IDA) in SGD". However, our results indicate its more precise localization as a component (transient or consistent) of the ribonuclease mitochondrial RNA processing (MRP) complex and small nucleolar ribonucleoprotein complex. Because the MRP complex is a ribonucleoprotein complex that performs the first cleavage in rRNA transcript processing, this prediction is consistent with one of its biological process annotations, rRNA processing [32], and the MIPS description on cellular complexes: "rRNA splicing". Compared with previous annotations to the cellular component category, our prediction descended two levels and identified the leaf node (nucleus-nucleolus-ribonuclease MRP complex). Recently, the component annotation of *IFH1 *was also assigned to nucleolus [31], which confirmed our results which were derived independently.

## Discussion

We have proposed a universal knowledge discovery framework to generate hypotheses for protein function annotation to the Gene Ontology biological process, molecular function and cellular component hierarchies in an automated fashion through mining of genome-scale data. Compared with previously published methods on protein function prediction, our method is distinctive in the following aspects:

(i) Flexibility in Prediction. Un-annotated proteins can be assigned to less specific or more precise annotations in all three hierarchies of the GO. A conservative estimation of precision for each prediction is also provided with these comprehensive descriptions of unknown protein function. This is in contrast to most other methods, in which proteins were assigned to a limited number of function categories [19,33] such as the MIPS or YPD (Yeast Proteome Database) [34], which are less detailed than GO, or to a single GO biological process hierarchy [21,22]. The universal and flexible characteristics of our protein function prediction, as demonstrated, for example, by aggressive prediction and normal prediction in this report, is significantly different from methods which use abstract categories in GO [24,25]. Although our results were consistently better using Normal Prediction, this was a reflection of the limited size of the data sets. As more data is accumulated, the statistics associated with Aggressive Predictions will improve and this prediction method will become more valuable.

(ii) Integration of Evidence Data. GMUP combines a much wider range of experimental data than previous analyses. This provides the opportunity to make new predictions based on more complex relationships. This strategy is universal to protein function prediction on any branch of GO and, in theory, it could be applied to other types of hierarchical ontologies beyond the Gene Ontology. Furthermore, it might also be used as an evaluation tool to study reliability and prediction capabilities of various data sources.

(iii) Architecture of the Model. We have made our predictions based on 16 sources of evidence data. However, since the evidence-pair database provides the abstraction layer by which all datasets can be related to each another, our method can easily accommodate additional datasets as they become available. For instance, nucleic acid sequence order information might provide specific contributions to the prediction of molecular function category in the GO.

## Conclusion

In summary, we have described a universal mapping strategy, in which protein-pair generation serves as a bridge from heterogeneous experimental evidence to knowledge representation. We have successfully applied the technique to make a number of novel predictions for genes associated with ribosomal function and which provide some insights into the mechanism and modes of interactions that are taking place. As more experimental evidence accumulates, the system can generate more comprehensive insights of how a certain biological process is implemented by the coordination of a group of proteins. This implementation is demonstrated by showing where the protein exists (cellular component and complex), in what manner it operates (molecular function), and with what components it collaborates (protein-protein interaction). The ongoing development of knowledge representation systems such as hierarchical ontology and their interaction with high-throughput experiments are becoming increasingly important in modern biology. The significance of these systems is not only in their ability to bridge gaps between hypotheses and the supporting data, but is also based on the potential to model complex biological systems based on well-structured hierarchical ontologies. The architecture of GMUP readily lends itself to the inclusion of additional data sources and we propose to add more features and datasets for optimizing the predictive performance. Additionally, we plan to investigate improving the accuracy of predictions by replacing the Neural Network with a Support Vector Machine (SVM). SVMs often obtain a greater success rate for this type of classification problem and can be easier to analyze theoretically. Finally, we also plan to incorporate weighting of GO assignments to reflect the accuracy of such assignments. The GO uses a series "evidence code" to represent the different source of annotation, such as "IDA: Inferred from Direct Assay", "IEA: Inferred from Electronic Annotation" etc. In the current study we used all such evidence without regard to source. By incorporating this data, we can filter the annotation data according to evidence-code and set criteria to adapt different reliable part of GO annotation to investigate its effects on protein prediction precision and sensitivity.

## Methods

### Selection and preparation of evidence data

We collected sixteen different biological evidence data from various types of high-throughput studies as follows. (i) Gene expression profiles from cell-cycle progression [1], the Rosetta Compendium [2], and MAPK signal transduction [3]. These data were downloaded from the ExpressDB RNA Expression Database [35]. (ii) Protein interaction data included physical and genetic interaction data in the MIPS database [36], Ito's data [37], Uetz's data [38], and interactions integrated in the Database of Interacting Proteins [39]. (iii) Protein complex data including the Biomolecular Interaction Network Database [40], MIPS complex data [41], and the High-Throughput Mass Spectrometric Protein Complex Identification (HMS-PCI) data [8]. (iv) Other in-silico analysis results, such as functional links between proteins generated by analysis of gene fusions and phylogenetic patterns in the Predictome database [42] were also included. Another data source was the co-expression data measured at the mRNA level ("SynExpress"), computationally predicted interactions ("In-silico"), and genetic interaction data ("Genetic") identified by C. Von Mering [43]. Datasets which do not have URLs listed in the references were obtained by submitting an enquiry directly to the original authors or downloading from the supporting web site for Deng's paper [44]. We use genes and proteins interchangeably. ORF naming is according to the SGD Gene Nomenclature Conventions [45].

We referred to these data as the evidence data. The selected data were chosen to represent experimental and predictive evidence from a diverse range of perspectives, as opposed to a data from a single related source of evidence.

**Figure 6:**
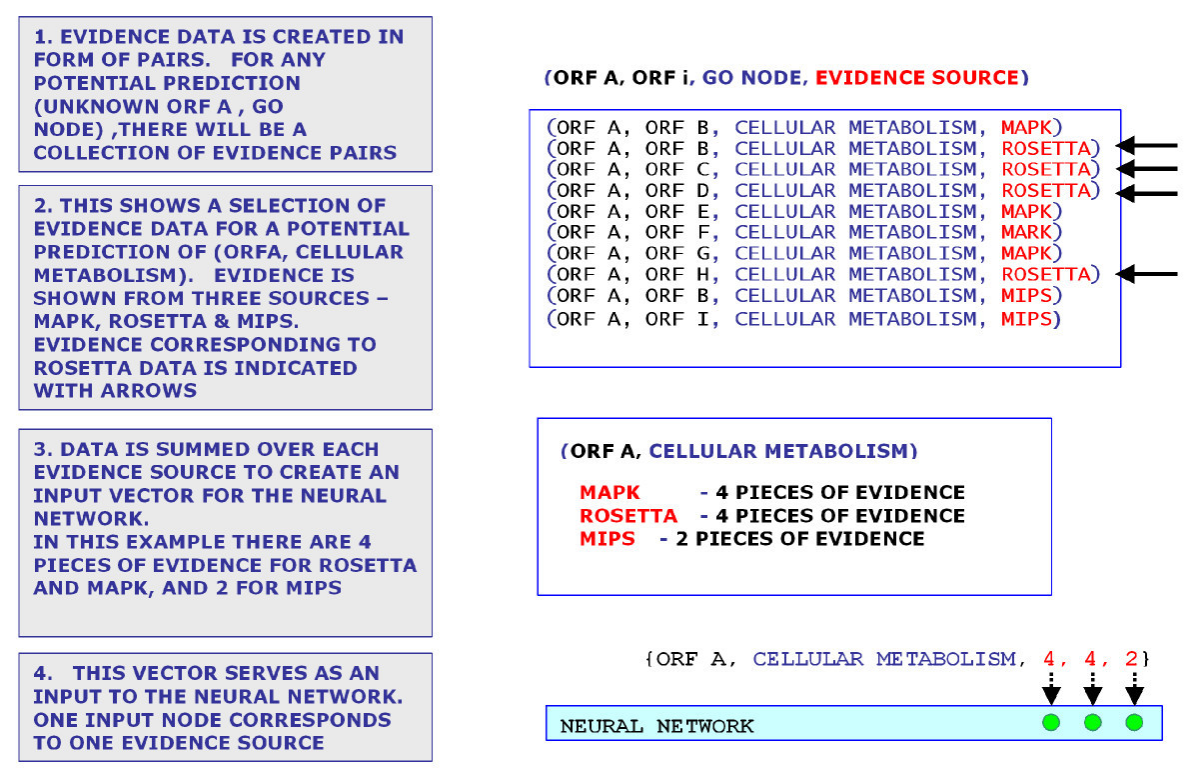
**Steps in the creation of the evidence vector for input to the neural network**. Pairs are created within each dataset (see **Methods**). Once the complete set of pairs have been generated and input to the pairs database, a query may be run for each ORF to identify the associated pairs and create an Evidence Vector for input to the neural network. Since the data comprises 16 distinct sets, this will create a 16 element vector.

To prepare the data for use with the network, we represented them as interacting pairs of ORFs in the format (*ORF*_*i*_, *ORF*_*j*_, *Predicted GO Node*, *Evidence Source*). Some data sources, such as the *Protein Interaction Data*, were already in the form (*ORF1 interaction with ORF2*) so evidence data could be formed directly from this information. We prepared the remaining evidence as follows (Fig. 6):

#### Gene expression data

The raw data was in the form of an n by m matrix which represents the response of n ORFs to m conditions, with each row in the matrix corresponding to the expression data for one ORF. For each gene expression dataset such as Rosetta, we calculated the Pearson coefficient of pairwise combination of any two ORFs over the m conditions, then took the 30,000 highest scoring pairs in each gene expression dataset.

#### Protein complex data

The raw data is in the form *Complex *(*ORF1*, *ORF2*, *ORF3*). We generated evidence data from all possible pair combinations from each complex.

#### All other data sources

Format was the same as for protein interaction data, so we formed evidence data in the same manner.

Once we had converted the data to evidence format, we stored it in a SQL "Evidence-Pair" database to simplify the process of integrating the evidence data with the annotation data (Table 2, see *Consolidation of Data Sources *below).

### Preparation of *yeast *ORF data

The number of hypothetical open reading frames (ORFs) for protein encoding genes in strain S288c of the yeast genome, including verified, uncharacterized and dubious, is currently estimated to be 6569. Of these, there are 6167 ORFs which have both a standardized name according to the SGD nomenclature and at least one associated annotation in the 21/4/2004 release of the GO index. Within this set of 6167 ORFs, there were 3863, 3235 and 3998 ORFs which had a valid annotation in the Biological Process, Molecular Function and Cellular Components subcategories respectively.

### Consolidation of the data sources

Each generated evidence pair (ORF_i_, ORF_j_) represents support for a particular annotation for ORF_i _(Table 3). For example, if we have predictions (ORFA, ORFB) and (ORFA, ORFC) where both ORFB and ORFC share the same GO annotation *G*, then we have two pieces of evidence which support the mapping of ORFA to GO annotation *G*. The evidence vector was generated for each ORF_i _as follows. From each data source *D*_d _(*d *= 1 to 16) we selected all occurrences of ORF_i _within each dataset to generate the evidence count for that source. We then combined the resulting 16 sums into a 16 element vector which served as input to our neural network (Fig. 6). As a consequence, some ORFs will have multiple potential function assignments/predictions, which refer to different evidence counts. We generate and validate each of these multiple predictions in turn to generate a set of predictions for that ORF. If these multiple prediction points to GO node which have parent relationship in GO graph, it would strengthen the reliability of either predictions thus our precision estimation is conservative.

For each of the 6167 ORFs with both SGD and GO annotation we ran a SQL query to collect all the associated data from our Evidence Database. This evidence was then combined into an evidence vector for that ORF. An excerpt from a protein-pair table with the evidence summary for a single ORF is shown in Table 4. Each row in the table corresponds to a protein pair prediction with the associated GO annotation and its evidence source (Fig. 6, and [see Additional file 2]).

### Prediction model

To train the neural network we used the fact that each of the three vocabularies defined in the GO index may be represented by a directed acyclic graph. Any node in any of the three graphs represents a functional assignment – the broadest assignments are at the top of the graph and, as additional nodes are traversed through subsequent layers, a progressively more precise functional assignment is obtained. For the purpose of matching GO assignment between ORFs, we defined *Aggressive Prediction *and *Normal Prediction *modes as our two methods of assigning protein function (Fig. 1b). In *Aggressive Prediction *mode, we seek the most specific assignment possible, i.e., we seek a match between nodes at the lowest possible level, representing more explicit definition of function. Conversely, in *Normal Prediction *mode, we seek a match between higher level (less explicit) nodes. In this prediction mode, function assignments may not be so precise but two ORFs may be shown to belong to the same functional family. Although a match at a higher level node provides less specificity in a match it pools more assignment information to provide a (statistically) more reliable prediction.

To rate the quality of a GO function match between two ORFs *i *and *j*, we defined a scoring model called *Inherited Node Scoring*. This model was based on the *Variant Distance *VD defined as the distance between the predicted node, and the true node (i.e. the GO assignment in the SGD database). The Inherited Node Scoring model is shown in Fig. 1a. In this model, if the predicted node NP was the same as the assigned node NA then VD = 0. If NP is an ancestor of NA then we also set VD = 0 (based on the assumption that any protein associated with one node is implicitly associated with every ancestor of it). For other predictions such as "NP is a child of NA" (see Fig. 1a, nodes a and c) or "NP is a sibling of NA" (see Fig. 1a, nodes a and d) VD was assigned a positive value equal to the minimum distance between the two nodes. In this scoring scheme, VD can take any positive integer, but to reduce the network to a two state classification system any positive value of VD was set to 1. This also had the effect of producing more conservative predictions since the network was trained to only recognise ancestral relationships between nodes sharing the same leaf of a graph as acceptable true matches.

### Neural network topology and training

We created a three layer back propagation neural network using MatLab. The Input Layer consisted of 16 nodes, one for each evidence source, and we prepared data as described above (see *Selection and Preparation of Evidence Data*). For training, we scored the quality of an ORF pair using the Inherited Node Scoring model with a score taking the value 0 or 1. For evaluation and prediction, the single output for the neural network was the variable *nnout *which could take a non integer value between 0 and 1. We calculated the variant distance *VD*_*ij *_between the two ORFs specified in the Evidence Vector according to whether *nnout *was greater than a threshold k, which could be adjusted from 0 to 1 at run time and controlled the precision and number of predictions made by the network. For example, a value of *k *close to 1 would exclude all but the most reliable predictions but provide very little information regarding function prediction. On the other hand, a value of *k *close to 0 would yield more predictions but with less reliability.

To evaluate network performance we followed the method of Kohavi [46] and defined the confusion matrix [see Additional file 12].

Where

*a *(*TN*, *true negative*) is the number of correct predictions that an instance is negative,

*b *(*FP*, *false positive*) is the number of incorrect predictions that an instance is positive,

*c *(*FN*, *false negative*) is the number of incorrect of predictions that an instance negative

d (*TP*, *true positive*) is the number of correct predictions that an instance is positive.

We then calculated the Precision and Sensitivity according to

*Precision *= *d*/(*b*+*d*)

*Sensitivity *= *d*/(*c*+*d*)

and plotted a graph of Precision vs Sensitivity by varying the threshold *k*. We optimized the network by seeking a maximum value for the area *Az *under the Precision/Sensitivity curve.

We used the Levenburg-Marquardt algorithm [47] for training and the matlab *Tansig *and *Poslin *functions as the transfer (activation) functions for the input and output layers respectively.

### Model validation and parameter optimization

Within this set of 6167 ORFs, there were 3863, 3773 and 3998 which had a valid annotation in the Biological Process, Molecular Function and Cellular Components subcategories respectively. We used a different network for each of the three GO vocabularies and one for each prediction model so that a total of 6 networks were trained, optimized and tested. For training and evaluation of our network, we randomly divided this entire set of annotated ORFs into training and evaluation categories in the ratio of 9:1. Thus the greater portion of the known annotation was used for training the network. We repeated this process 20 times to generate additional random datasets which could be used to investigate network behaviour.

The area *Az *under the Precision-Sensitivity curves was used as an evaluation of the network performance (see *Neural Network Topology and Training *above). Network performance can be affected by issues such as imbalanced data and the configuration of the network. To try and evaluate the impact of these effects we used cross-validation to compare the performance of the network with different parameters.

To compensate for imbalanced output data we applied a set of correction parameters *P *which scaled the data either by under-sampling (for overrepresented data) or over-sampling (for underrepresented data). For example, to correct for underrepresented data, we would oversample that set by a factor P. Conversely, overrepresented data would be undersampled by a factor 1/P. To find the optimal scaling factor, the network was retrained for a set of scaling parameters (*P *= 2^n^; *n *= 1, 2, 3 .. 6) to identify the value of P which produced the best network performance. The source codes for cross validation, neural network training and random data sampling were also supplied [see Additional file 11].

We also investigated the effect of adjusting *N*_*H*_, the number of nodes in the hidden layer, and retrained the network for 4 <*N*_*H *_< 16 to examine the effect on network performance. We found that the optimal network performance was obtained for 8 nodes in the hidden layer [see Additional file 3].

### Modification of GO ontologies

The topology of the GO graphs is extensive and the yeast ORF data only maps to a small portion of any graph. In the Normal Predictions Model, in order to improve the statistics of the evidence vector we "pruned" the GO graphs to keep only the nodes that were highly represented by the evidence data. We defined the root node of each graph to be level 1, the subsequent layer as level 2 and so on. After pruning we had a total of 195 nodes consisting of 71 nodes (level 4) in the BP category, 84 nodes (level 3) in the MF category, and 40 nodes (level 4) in the CC category and these were used as the targets of normal predictions.

For presentation of the results in figure 2, we selected the nodes which had the greatest number of associated predictions for gene function to get the top 30 nodes in the list for the BP, MF, and CC categories. If two of these nodes had a parent-child relationship, we deleted the parent node from the list. This left 14 BP nodes, 8 MF nodes, and 5 CC nodes. Finally, we reordered the evidence data of all the ORFs that were annotated into these nodes as independent datasets and calculated their precision-sensitivity curves (Fig. 2) Applying a standard tenfold cross validation was repeated 20 times to calculate the mean of the area under the curve.

### Prediction of uncharacterized ORFs

To assign functions to uncharacterized ORFs, we input the associated ORF-evidence vector to the parameter-optimized neural network, and calculated the corresponding *nnout*. To calculate the precision associated with each prediction, we selected one set of the high-level parent nodes (the same set as used in the normal prediction model) and generated the *nnout-precision *function curve for each node in the set. This was done by adjusting the threshold value *k *and iterating through the predictions associated with that node to see how the predictions were classified. For each point in the curve, we incremented *k *and repeated the iteration. (see *Neural Network Topology and Training *Above). We then fitted a polynomial of degree 6 to the resulting curve. To ensure robustness, we selected GO nodes that contained more than 50 positive predictions. The fitted *nnout-precision *curve was then used to calculate the corresponding precision of a candidate prediction assigning protein to GO node.

## Authors' contributions

JX was responsible for design and conceptualization, took part in implementation, and drafted the manuscript. SR took part in analysis and drafted the manuscript. KL assisted with development of several data processing procedures. YL and SC participated in revision of the draft critically.

## Supplementary Material

Additional File 1 **CompMIPS**. A complete list for comparison of our predictions with their MIPS description. Click here for file

Additional File 2 **Fig S1**. Illustration of formation of Evidence Vector from Protein Pair Database. Click here for file

Additional File 3 **Fig S2**. The effects of number of nodes in the hidden layer on neural network performance (Several subplot have missing bars since the memory used exceeded the limitations of Matlab). Click here for file

Additional File 4 **Fig S3**. Validation of our predictions with new released GO annotation data according to Inherited Scoring Model (The red "+" in the center of circle indicated the x and y axis, and the radius of circles is proportion to frequency of Distance Score for each GO nodes). Click here for file

Additional File 5 **DataDesp**. The description of all data sets and naming guide. Click here for file

Additional File 6 **ReleaseA_BP**. [see Additional file 5] for detailed description. Click here for file

Additional File 7 **ReleaseA_MF**. [see Additional file 5] for detailed description. Click here for file

Additional File 8 **ReleaseA_CC**. [see Additional file 5] for detailed description. Click here for file

Additional File 9 **ReleaseB_known**. [see Additional file 5] for detailed description. Click here for file

Additional File 10 **ReleaseB_Unknown**. [see Additional file 5] for detailed description. Click here for file

Additional File 11 **Source**. Source codes for cross validation, neural network training and random data sampling. Click here for file

Additional File 12 **Conf_matrix**. Illustration of the confusion matrix. Click here for file

## Figures and Tables

**Table 1:** Comparison of our predictions on gene function with their MIPS description ([see Additional file 1] for full data set)

**ORF/Gene**	**Ontology**	**Prediction**	**MIPS description**
ASC1	BP	Biosynthesis	PROTEIN SYNTHESIS\ribosome biogenesis; PROTEIN SYNTHESIS\translation
ASC1	CC	cytosolic small ribosomal subunit; ribonucleoprotein complex	Cytoplasm
ASC1	MF	structural constituent of ribosome	GTP-binding proteins\trimeric GTP-proteins\beta subunits
YGL068W	BP	fatty acid metabolism; protein metabolism	BIOGENESIS OF CELLULAR COMPONENTS\mitochondrion; PROTEIN SYNTHESIS\ribosome biogenesis\ribosomal proteins
YGL068W	CC	mitochondrial large ribosomal subunit; ribonucleoprotein complex	Mitochondria

These ORFs were selected from among the 78 predictions which have their equal annotation in MIPS database and their anticipated precision are larger than 80%.

**Table 2:** Illustration of protein-pair with evidence source

**ORF1**	**ORF2**	**Evidence source**
YAL001C	YBL002W	Pearson correlation @ MAPK microarray
YAL001C	YBR123C	complex @ MIPS
YAL001C	YBR123C	protein interaction @ DIP
YAL001C	YBR160W	Pearson correlation @ MAPK microarray
YAL001C	YDR362C	complex @ MIPS
YAL001C	YDR362C	genetic interaction @ MIPS
YAL001C	YDR362C	physical interaction @ MIPS
YAL002W	YAL011W	Pearson correlation @ cell cycle microarray
YAL002W	YBL030C	complex @ BIND
YAL002W	YBL030C	HMS-PCI complex
YAL002W	YBL094C	Pearson correlation @ cell cycle microarray
YAL002W	YBL096C	Pearson correlation @ cell cycle microarray
YAL002W	YKR026C	protein interaction @ DIP

**Table 3:** Excerpt from protein-pair database sorted by unknown ORF

**ORF1**	**Predictive GO term**	**Evidence source**	**ORF2**
YGL068W	protein biosynthesis	complex @ MIPS	YBL038W
YGL068W	protein biosynthesis	complex @ MIPS	YBR122C
YGL068W	protein biosynthesis	complex @ MIPS	YBR268W
YGL068W	35S primary transcript processing	In-silico interaction data @ C. Von Mering et al	YHR148W
YGL068W	aerobic respiration	complex @ MIPS	YDR116C
YGL068W	fatty acid metabolism	complex @ MIPS	YEL050C
YGL068W	fatty acid metabolism	In-silico interaction data @ C. Von Mering et al	YEL050C

**Table 4:** Illustration of evidence combination vector

ORF	**Predictive GO term**	**Evidence combination**					
		
		**Cell cycle**	**Rosetta**	**MAPK**	**MIPS physical**	**MIPS complex**	**...**
YAL001C	RNA polymerase III transcription factor activity	0	0	30	1	4	...
YAL001C	transcription factor TFIIIC complex	0	0	30	1	4	...
YAL001C	transcription initiation from Pol III promoter	0	0	60	2	8	...
YAL002W	late endosome to vacuole transport	18	0	0	0	0	...
YAL002W	membrane fraction	3	0	0	0	0	...
YAL003W	Ribosome	44	28	0	6	10	...
YAL003W	translation elongation factor activity	44	28	0	6	10	...
YAL003W	translational elongation	44	28	0	6	10	...
YAL005C	ATPase activity	16	1	2	4	0	...
YAL005C	cell wall (sensu Fungi)	16	1	2	4	0	...

**Table 5:** Fragments of data sources from functional links between proteins in Predictome database

**ORF A**	**ORF B**	**METHOD NAME**
YER103W	YJL073W	Phylogenetic Pattern
YER103W	YMR161W	Phylogenetic Pattern
YER065C	YIR031C	Phylogenetic Pattern
YPR189W	YPL157W	Gene Fusion
YPR198W	YBR041W	Gene Fusion
YPR193C	YHR074W	Gene Fusion
