# Supplementary figures and images for: Genome wide prediction of protein function via a generic knowledge discovery approach based on evidence integration
Source: BMC Bioinformatics. 2006 May 25;7:268. doi: 10.1186/1471-2105-7-268 (PMC1481625; doi:10.1186/1471-2105-7-268)

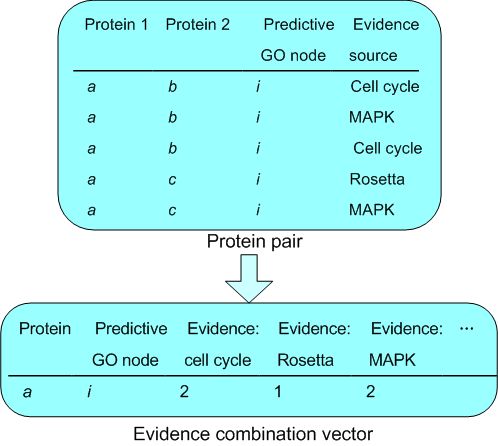

Supplement: Additional File 2 — Fig S1. Illustration of formation of Evidence Vector from Protein Pair Database. [file 1471-2105-7-268-S2.jpeg]

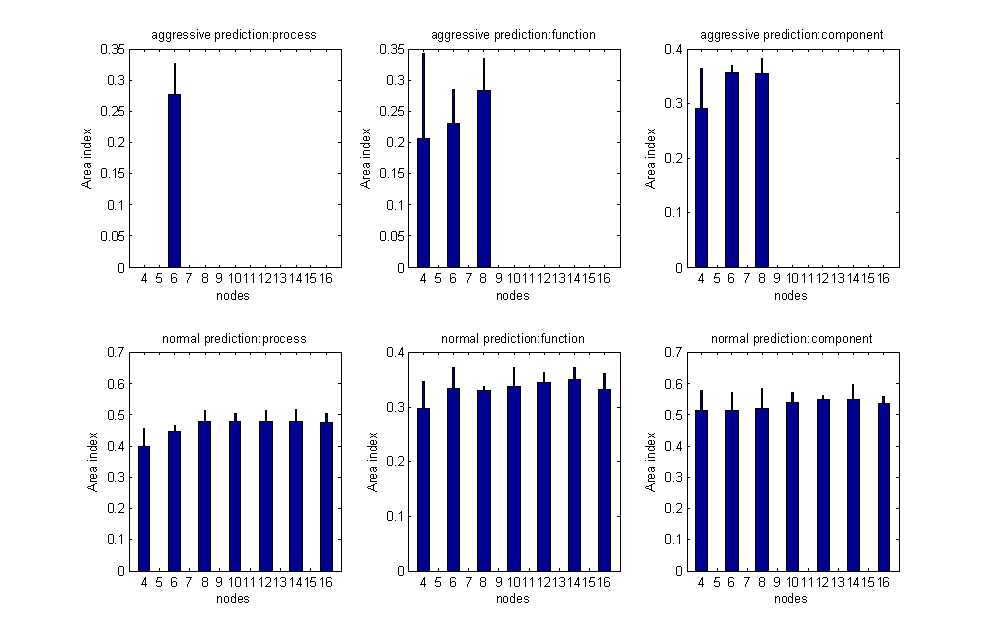

Supplement: Additional File 3 — Fig S2. The effects of number of nodes in the hidden layer on neural network performance (Several subplot have missing bars since the memory used exceeded the limitations of Matlab). [file 1471-2105-7-268-S3.jpeg]

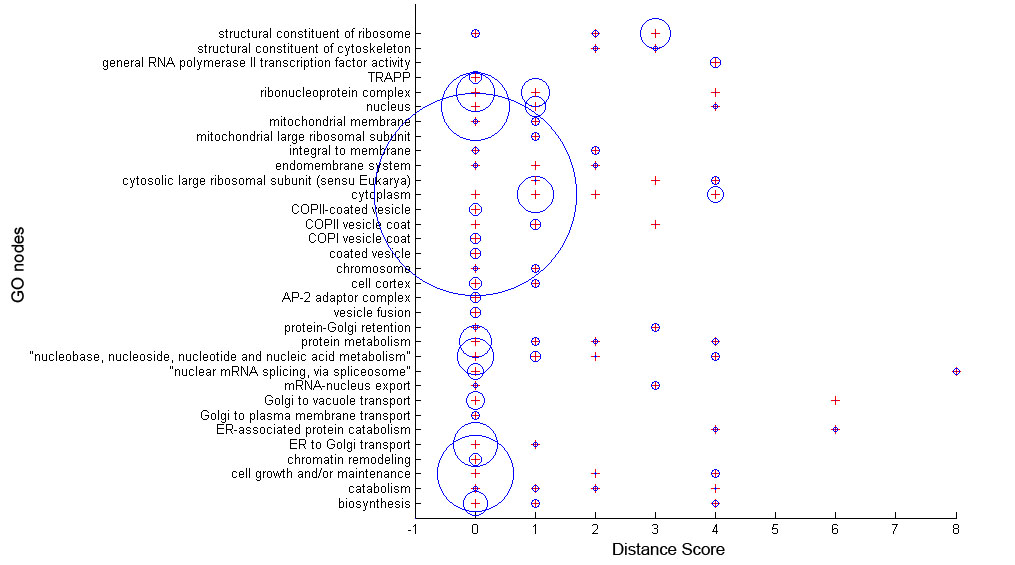

Supplement: Additional File 4 — Fig S3. Validation of our predictions with new released GO annotation data according to Inherited Scoring Model (The red "+" in the center of circle indicated the x and y axis, and the radius of circles is proportion to frequency of Distance Score for each GO nodes). [file 1471-2105-7-268-S4.jpeg]

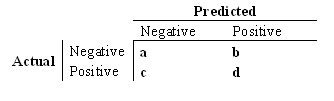

Supplement: Additional File 12 — Conf_matrix. Illustration of the confusion matrix. [file 1471-2105-7-268-S12.jpeg]
