# Supplementary material for: Genome wide prediction of protein function via a generic knowledge discovery approach based on evidence integration
Source: BMC Bioinformatics. 2006 May 25;7:268. doi: 10.1186/1471-2105-7-268 (PMC1481625; doi:10.1186/1471-2105-7-268)
Supplement: Additional File 5 — DataDesp. The description of all data sets and naming guide. [file 1471-2105-7-268-S5.doc]

**All data sets and naming guide**

**GO annotation Release A**

- - Date: 04/21/2004
  - Total ORFs:6167
  - Total known ORFs
    - BP 3863
    - MF 3235
    - CC 3998
  - Total Unknown ORFs
    - BP 2304
    - MF 2932
    - CC 2169
  - ANN Predictions on this dataset
    - Data file
      - ReleaseA_BP.xls
      - ReleaseA_MF.xls
      - ReleaseA_CC.xls
    - Statistics (conservative precision > 0.3):
      - BP: 1980 ORFs
      - MF: 836 ORFs
      - CC: 1969 ORFs

**GO annotation Release B**

- - Date: 09/27/2005
  - Total ORFs : the same as Release A (6167)
  - Total ORFs That have been Annotated Since Release A
    - BP 4009
    - MF 3292
    - CC 4099
  - New valid annotation comparing this release to release A:
    - BP 4009-3863=146
    - MF 3292-3235=57
    - CC 4099-3998=101
  - Total Unknown ORFs
    - BP 2158
    - MF 2875
    - CC 2068
  - ANN Predictions for these new released ORFs (which ORFs is unknown in release A but have annotation in release B)
    - Data file: ReleaseB_known.xls
    - Brief Notes: In this high quality predictions (precision >=0.8), there are 154 ORFs which have new released GO annotation. We compare this 472 predictions with their corresponding GO annotation (see Additional file 4:Fig S3.jpg)
  - ANN Predictions for unknown ORFs
    - Data file: ReleaseB_Unknown.xls
    - Brief notes: For ORFs which had no GO annotation, we made 624 predictions for 232 ORFs in an acceptable precision level (>=0.6).
